# Video-thoracoscopic resection of lung metastases using the Nd:YAG Laser LIMAX^® ^120

**DOI:** 10.1186/1749-8090-10-S1-A22

**Published:** 2015-12-16

**Authors:** Andreas Kirschbaum, Christian Meyer, Detlef K Bartsch

**Affiliations:** 1 Department of visceral-, thoracic- and vascular surgery, University Hospital Marburg, D-35033 Marburg, Germany

## Background/Introduction

In general, resection of pulmonary metastases by laser is performed by means of a thoracotomy. As an alternative, we developed a video-assisted technique that allows palpating of the entire lung as well as resection of the pulmonary metastases by laser.

## Aims/Objectives

We report our first experiences with this technique.

## Method

10 patients (7 ♂, 3 ♀; age: 22 - 83 years) were diagnosed with pulmonary metastases of different primary tumours. The procedure begins with creation of a mini-thoracotomy at the level of the 5th ICR, approximately 4 cm in length. No rib retractor is applied, but wound protection film (Applied Medical, CA, USA) is used. Through a basally applied trocar, the video thoracoscope is introduced. Then the pulmonary ligament is cut and video-assisted radical mediastinal lymphadenectomy is performed. The entire lung is then palpated through the mini-thoracotomy. The respective lung metastases are identified and resected under thoracoscopic control with the Nd:YAG laser LIMAX^® ^120 (Gebrüder Martin & Co. KG, Tuttlingen, Germany). To this end, under visual control a hand piece with a focus distance of 3 cm is introduced at a laser power of 80 watts. Deeper parenchymal lesions are closed under video-thoracoscopic control with a monofilament suture (PDS 4-0). Usually only one intercostal drainage with additional holes is necessary. The mini-thoracotomy is closed by one pericostal suture.

## Results

The mean duration of the surgical procedure was 90 minutes. On average, 2 lung metastases per patient were removed. Histologically, the resection margins were always clear. The postoperative courses were uncomplicated; the intercostal drainages applied could be removed median on the 4th (range 3 to 6) postoperative day. All patients could be discharged symptom-free between the 5th and 6th postoperative day. Control by thoracic CT scanning after 3 months showed no evidence of pulmonary metastases.

## Discussion/Conclusion

Using the Nd:YAG laser LIMAX^® ^120, pulmonary metastases can be removed safely and in sano by a video-assisted procedure. Thoracotomy can thus be avoided. Nevertheless, the surgical technique described by us meets the requirements of modern metastasis surgery.

